# Use of NHFOV vs. NIPPV for the respiratory support of preterm newborns after extubation: A meta-analysis

**DOI:** 10.3389/fped.2022.1063387

**Published:** 2023-01-11

**Authors:** Zhaojun Mei, Li Ming, Zhifeng Wu, Yong Zhu

**Affiliations:** ^1^ Luzhou Maternal and Child Health Hospital, Luzhou Second People's Hospital, Luzhou, China; ^2^ Department of Pediatrics, Xinqiao Hospital, Army Medical University, Chongqing, China; ^3^ Department of Pediatric Center, University-Town Hospital of Chongqing Medical University, Chongqing, China

**Keywords:** noninvasive high-frequency oscillatory ventilation, nasal intermittent positive-pressure ventilation, preterm infants, respiratory support, meta-analysis

## Abstract

**Objectives:**

This meta-analysis evaluated and compared the efficacy and safety of noninvasive high-frequency oscillatory ventilation (NHFOV) and nasal intermittent positive-pressure ventilation (NIPPV) for preterm newborns after extubation.

**Methods:**

We searched the PubMed, Cochrane Library, EMBASE, Web of Science, CNKI, Wanfang and VIP databases from inception to August 28, 2022. Randomized controlled trials (RCTs) that evaluated and compared the efficacy and safety of NHFOV and NIPPV in newborns were included in the review and meta-analysis, which followed the Preferred Reporting Items for Systematic Reviews and Meta-Analyses (PRISMA) reporting guidelines.

**Results:**

Eight studies involving 1,603 patients were included. Compared with NIPPV, NHFOV could reduce the reintubation rates (RR = 0.68, 95% CI 0.53, 0.86, *P* = 0.002). Subgroup analysis showed that the significant difference was found in reintubation rates within 72 h (RR = 0.48, 95% CI 0.32, 0.73, *P* = 0.0005). NHFOV also could decrease the duration of non-invasive ventilation (standard mean difference (SMD) = −1.52, 95% CI −2.58, −0.45, *P* = 0.005). However, all included studies had a high risk of bias, and the overall quality of the evidence of the outcomes was low or very low.

**Conclusion:**

In our study, compared with NIPPV, NHFOV seems to reduce the reintubation rates without increasing adverse outcomes. Nevertheless, definite recommendations cannot be made based on the quality of the published evidence.

## Introduction

Preterm infants are prone to various conditions because of their immature organs. Respiratory failure related to organ immaturity is the most common cause of death in preterm infants. Invasive mechanical ventilation (IMV), which has been widely used in past decades to support preterm infants with respiratory diseases. Although lifesaving, IMV is an important risk factor in the development of many complications such as air leaks, ventilator-associated lung injury, and bronchopulmonary dysplasia (BPD) (1). In the past decade, the practice of prompt weaning and early extubation to non-invasive respiratory support has been the focus and ultimate goal (2). However, some preterm infants need to be reintubated after a trial of extubation. An international survey found that 43% of experts believed that extubation failure is an independent risk factor for increased mortality and morbidity (3).

Continuous positive airway pressure (CPAP) is the most commonly used respiratory support in clinical practice. CPAP significantly reduced the need for IMV, but failure rates of almost 50% have prompted neonatologists to seek more effective noninvasive ventilation modalities (4). The latest Cochrane systematic review (5) suggest that nasal intermittent positive pressure ventilation (NIPPV) reduces the incidence of extubation failure and the need for re-intubation within 48 h to one week more effectively than nasal CPAP, but does not substantially reduce chronic lung disease and mortality. The synchronisation may be important in delivering effective NIPPV. In practice, however, synchronization is difficult to achieve due to leaks, high respiratory rate, low tidal volume, and irregular breathing pattern. Noninvasive high-frequency oscillatory ventilation (NHFOV) is an unconventional noninvasive ventilation mode, which theoretically provides the advantages of HFOV (no need for synchronization, high CO_2_ removal, lower volume/barotrauma) and nasal continuous positive airway pressure (noninvasive, increased in functional residual capacity allowing oxygenation to improve) (6). Therefore, this method is regarded as a possible improvement over continuous positive airway pressure. Recent several systematic reviews and meta-analyses (7, 8) have shown that the relative risk of intubation was decreased with NHFOV in comparison with nasal CPAP in preterm infants with respiratory distress syndrome. Both NIPPV and NHFOV produced significantly greater improvements in respiratory support of preterm newborns than CPAP. However, to date, evidence for effectiveness between NHFOV and NIPPV at reducing the rate of reintubation was still unknown, and there is no relevant meta-analysis at present.

Hence, we chose to meta-analyze the impact of the NHFOV and NIPPV on respiratory support among preterm neonates after extubation. This may help clinicians determine the best strategy for respiratory support of preterm newborns while identifying knowledge gaps requiring further research.

## Materials and methods

We conducted a systematic review and meta-analysis following the Preferred Reporting Items for Systematic Reviews and Meta-Analyses (PRISMA) guidelines (9).

### Search strategy

Two authors independently searched the PubMed, Cochrane Library, EMBASE, Web of Science from inception to August 28, 2022, using the following search terms: ((Infant OR newborn OR neonat* OR premature OR very low birth weight OR low birth weight OR VLBW OR LBW) AND (Noninvasive High-Frequency Oscillatory Ventilation OR Noninvasive High Frequency Oscillation Ventilation OR Non-invasive high-frequency oscillatory ventilation OR NHFOV OR nHFV) AND (nasal intermittent positive pressure ventilation OR NIPPV OR nasal intermittent mandatory ventilation OR NIMV OR non-invasive positive pressure ventilation)). At the same time, two authors also searched the most commonly used and comprehensive Chinese scientific literature databases (CNKI, Wanfang and VIP databases). No language restrictions were applied. A third author was consulted for the authors' differences in opinion during the study selection process.

### Inclusion and exclusion criteria

All included studies met the following criteria: (1) randomized controlled trial (RCT); (2) the intervention group was given NHFOV and the comparison group was given NIPPV as post-extubation respiratory support; And (3) at least one of the following outcome parameters was reported. The primary outcome was the rate of reintubation. The secondary outcomes included: (i) the duration of non-invasive ventilation, (ii) total oxygen therapy time, (iii) length of hospital stay (LOS), and (v) adverse outcomes, including air leak, abdominal distension, BPD, intraventricular hemorrhage (IVH), retinopathy of prematurity (ROP), necrotizing enterocolitis (NEC), nasal injury, periventricular leukomalacia, and apnea. The exclusion criteria were as follows: (1) non-clinical studies, (2) research protocols, (3) duplicated reports or secondary or post-hoc analyses of the same study population, or (4) lack of sufficient information related to baseline or outcome data.

### Data extraction

Two authors used pre-designed tables to extract data independently from each of the eligible studies. Disagreements between the two investigators were resolved by discussion or consensus with a third author. We extracted the characteristics of each study and recorded the following data: first author, year of publication, study design, characteristics of the study population, sample size, and details related to the methodological quality and results. The numeric results, statistics used and *p* values were extracted for each outcome. We attempted to contact the author of the original report to obtain further details when any of the above information was unclear.

### Quality assessment and publication bias

We assessed the quality of the included trials based on the information in the methods section and supplementary materials about them. The quality of the RCTs was assessed using the Cochrane Collaboration's risk-of-bias tool for randomized trials (RoB) (10), which consists of six domains and allowances for any other bias, with risk-of-bias judgments for RCTs ranging from “high,” “unclear” to “low”. Two authors independently assessed the studies' quality and resolved disagreements through consensus.

We used funnel plots to assess publication bias, which was calculated using RevMan 5.3 software. The Egger regression test was used to measure funnel plot asymmetry and was calculated using Stata 12.0 (StataCorp LP, College Station TX, United States).

### Data synthesis and analysis

Review Manager (RevMan) version 5.3 (Copenhagen: The Nordic Cochrane Centre, The Cochrane Collaboration, 2014) was used to calculate the pooled estimates. Risk Ratio (RR) for dichotomous outcomes, the standardized mean difference (SMD) for continuous data and corresponding 95% confidence intervals (CI) were used for the analyses. The *I*^2^ statistic was used to evaluate the effect of heterogeneity on the pooled results (*I*^2^ > 50% indicated substantial heterogeneity). A fixed-effects model was used to pool data when the heterogeneity was not significant and a random-effects model was used when significant heterogeneity was identified. We conducted sensitivity analyses by omitting studies one by one in order to probe the impact of an individual study. A *p*-value < 0.05 was considered statistically significant.

### Evidence assessed

Two authors assessed the certainty of the evidence (also known as the quality of the evidence) using the Grading of Recommendations Assessment, Development and Evaluation (GRADE) (11) approach at the outcome level for each comparison between interventions. The certainty in the evidence could be high, moderate, low, or very low.

## Results

### Study inclusion and characteristics

**Figure 1:**
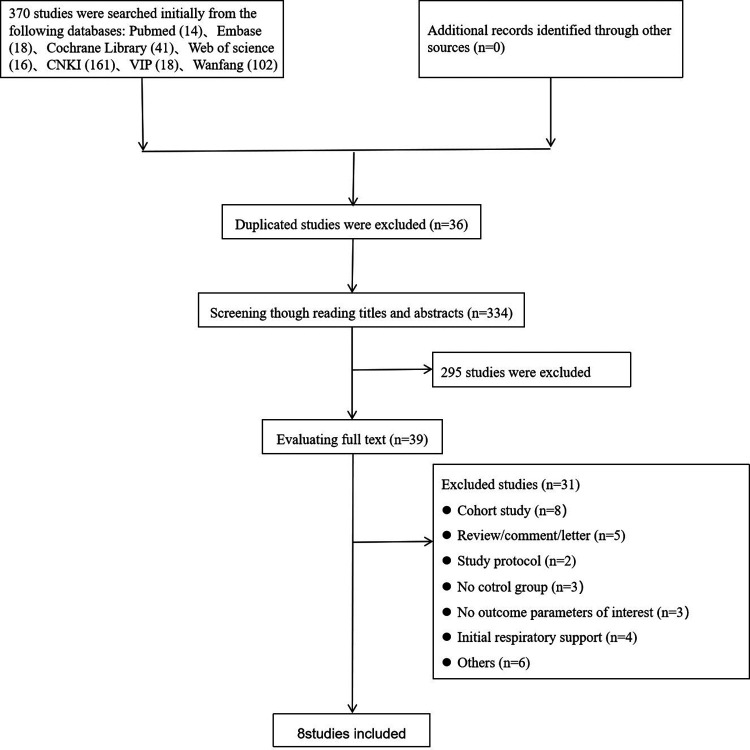
Flow diagram of the identification and selection of trails.

In the initial literature search (up to 28, August 2022), 370 studies were yielded. After removing duplicates, we screened the titles and abstracts of 334 studies and excluded 295 that did not meet our eligibility criteria. After evaluating the full text of the remaining 39 studies, we included 8 studies (12–19) in our meta-analysis (Figure 1). In total 1,603 participants of whom 799 received NHFOV for respiratory support, were included in our meta-analysis (Table 1). The publication dates of the RCTs ranged from 2019 to 2022, and 87.5% of the RCTs were done in China. Although, synchronized (patient-triggered) NIPPV seems more effective than NIPPV in improving the success of extubation, with a reassuring absence of relevant side effects. Synchronization is difficult to achieve and is often unavailable, so all RCTs included in our meta-analysis used non-synchronous NIPPV. All RCTs were compared at baseline, and there were no significant differences between the two groups in gestational age and birth weight.

**Table 1:** Characteristics of the 8 included trials that compared NHFOV with NIPPV.

Study	Country	Group	N	Male (n)	GA (wk)	BW (g)	Outcomes measures
Yan Zhuan 2021	China	NHFOV	45	29	28.6 ± 2.0	1039 ± 223	(1)(3)(4)(5)
		NIPPV	45	27	28.4 ± 2.2	1029 ± 230	
Xingwang Zhu 2022	China	NHFOV	480	296	29.4 ± 1.8	1317 ± 353.0	(1)(2)(3)(5)
		NIPPV	480	292	29.4 ± 1.8	1334 ± 366.0	
Yan Li 2021	China	NHFOV	45	22	29.0 ± 1.9	1118.9 ± 201.9	(1)(2)(3)(4)(5)
		NIPPV	47	25	28.9 ± 2.0	1088.5 ± 153.7	
Soutrik Seth 2021	India	NHFOV	43	24	32 (28 to 35)	1,500 (1,120 to 2140)	(1)(2)(5)
		NIPPV	43	24	31 (29 to 35)	1,495 (980 to 2214)	
Yanli Jia 2021	China	NHFOV	50	28	31.89 ± 1.42	1680 ± 350	(1)(2)(3)(5)
		NIPPV	50	29	31.77 ± 1.50	1650 ± 400	
Xiaozhan Huang 2021	China	NHFOV	65	34	32.95 ± 1.65	1728.92 ± 498.78	(1)(2)(5)
		NIPPV	65	33	32.89 ± 1.71	1733.33 ± 491.65	
Zhenyu Liang2019	China	NHFOV	21	15	30.86 ± 3.01	1472.34 ± 102.55	(1)(5)
		NIPPV	21	13	31.02 ± 3.23	1488.02 ± 105.63	
Zhu Wang 2019	China	NHFOV	50	21	29.7 ± 1.2	1270 ± 115	(1)(2)(3)(5)
		NIPPV	53	24	29.6 ± 1.4	1265 ± 120	

(1) Reintubation rates.

(2) The duration of non-invasive ventilation.

(3) Total oxygen therapy time.

(4) Length of hospital stay.

(5) Adverse outcomes.

### Methodological quality and risk of bias

**Figure 2:**
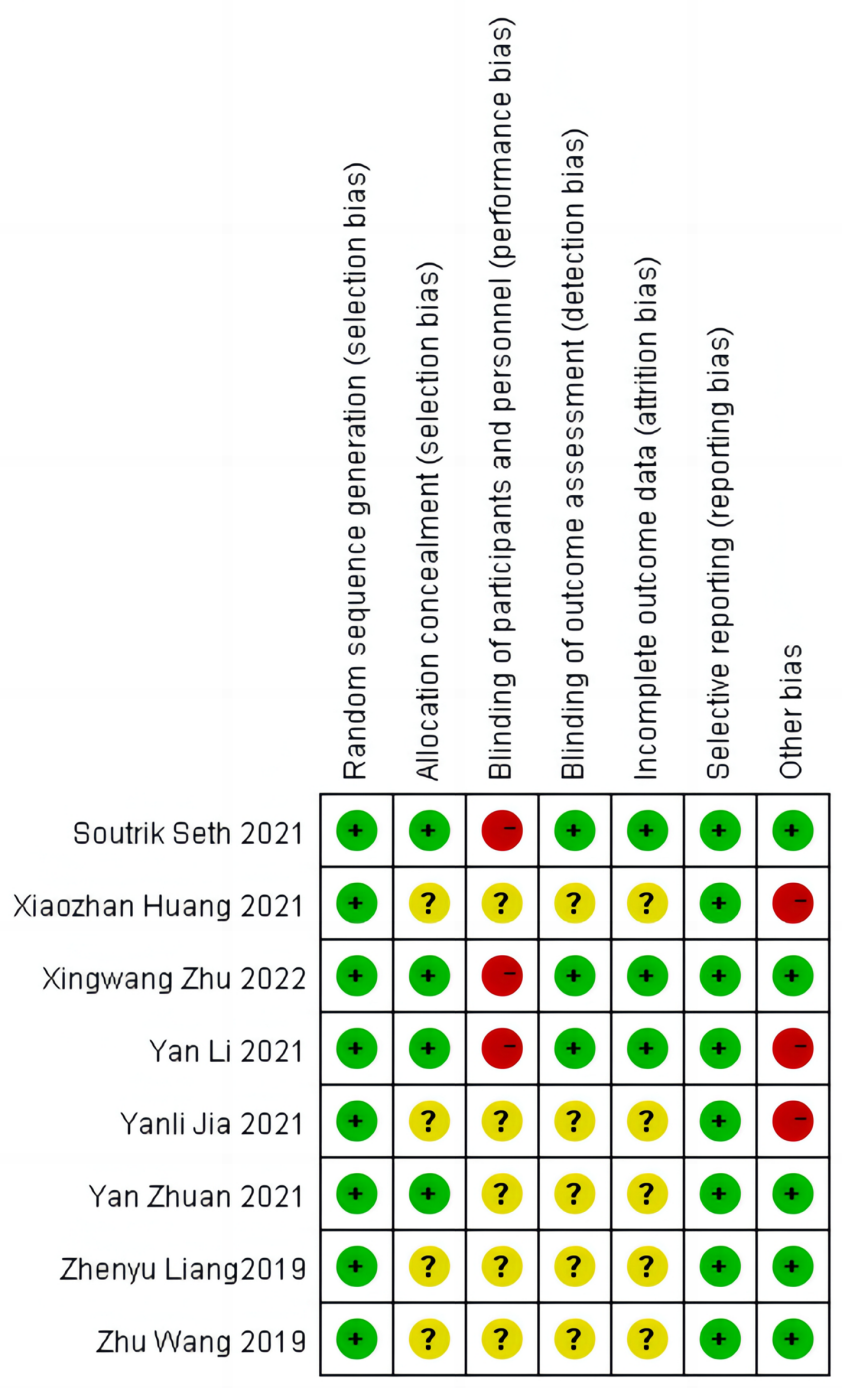
Risk of bias summary.

The RCTs were assessed using the Cochrane Collaboration risk of bias (RoB) tool. Adequate sequence generation was reported in eight studies (12–19), for them, a randomization sequence was generated using a computer-based random number generator. The method of allocation concealment was adequately reported in four studies. In the study of Soutrik Seth (16) and Yan Li (17), allocation concealment was done by using an opaque sealed envelope. In the study of Xingwang Zhu (12), allocation concealment was performed using a dedicated and secured website. The website generated the randomization, but the sequence was concealed from investigators at each of the participating sites. In the study of Yan Zhuan (12), the allocation was performed by a non-involved person. The infants and personnel could not be blinded due to the nature of the intervention and three studies were judged to have a high risk of bias due to performance bias; however, the outcome assessor was blinded, resulting in a low risk of bias due to detection bias. Three Studies demonstrated adequate follow-up of patients and accounted for any missing participants (12, 16, 17). In the remaining five studies, there were no reports of dropout of cases from randomization to the ascertainment, resulting in an unclear risk of bias due to attrition bias. In three studies (13, 14, 17), the definitions of some of the outcomes were not clear, the incidence of outcomes such as BPD can vary widely based on the definition. Therefore, they were considered to have a high risk of other biases. (Figure 2).

### Primary outcome

**Figure 3:**
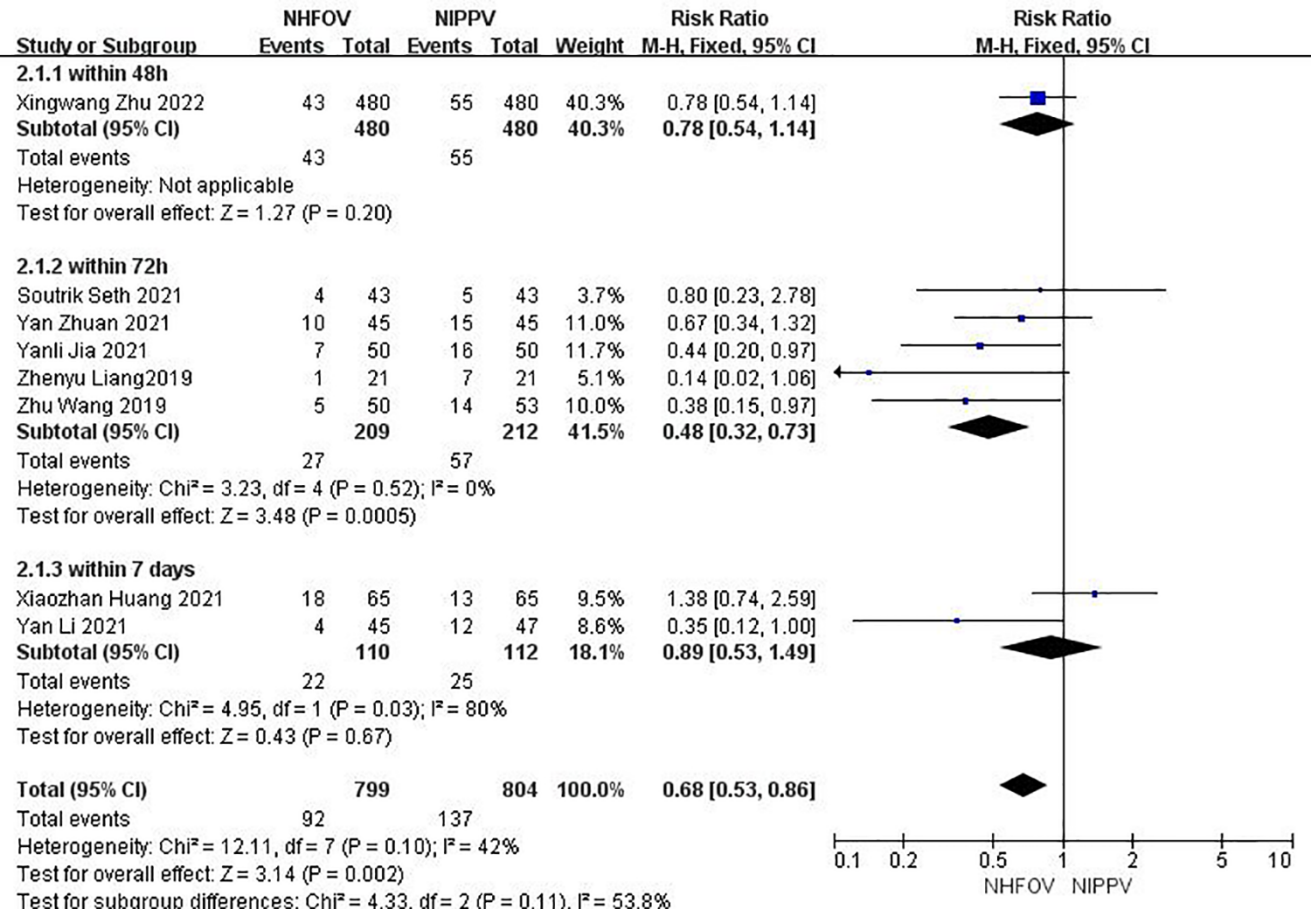
Results of the meta-analysis of reintubation rates; NHFOV, noninvasive high-frequency oscillatory ventilation; NIPPV, nasal intermittent positive-pressure ventilation.

Eight RCTs (12–19) that reported the reintubation rates. Significant difference was found in the reduction of reintubation rates between NHFOV and NIPPV (RR = 0.68, 95% CI 0.53, 0.86, *I*^2^ = 42%, *P* = 0.002; very low-quality evidence; Figure 3). Among them, one RCT that reported reintubation rates within 48 h found no significant differences between NHFOV and NIPPV (RR = 0.78, 95% CI 0.54, 1.14, *P* = 0.20; very low-quality evidence; Figure 3). Five RCTs that reported reintubation rates within 72 h found significant differences between NHFOV and NIPPV (RR = 0.48, 95% CI 0.32, 0.73, *I*^2^ = 0%, *P* = 0.0005; very low-quality evidence; Figure 3). Two RCTs that reported reintubation rates within 7 days found no significant differences (RR = 0.74, 95% CI 0.19, 2.89, *I*^2^ = 80%, *P* = 0.67; very low-quality evidence; Figure 3).

### Secondary outcomes

**Figure 4:**
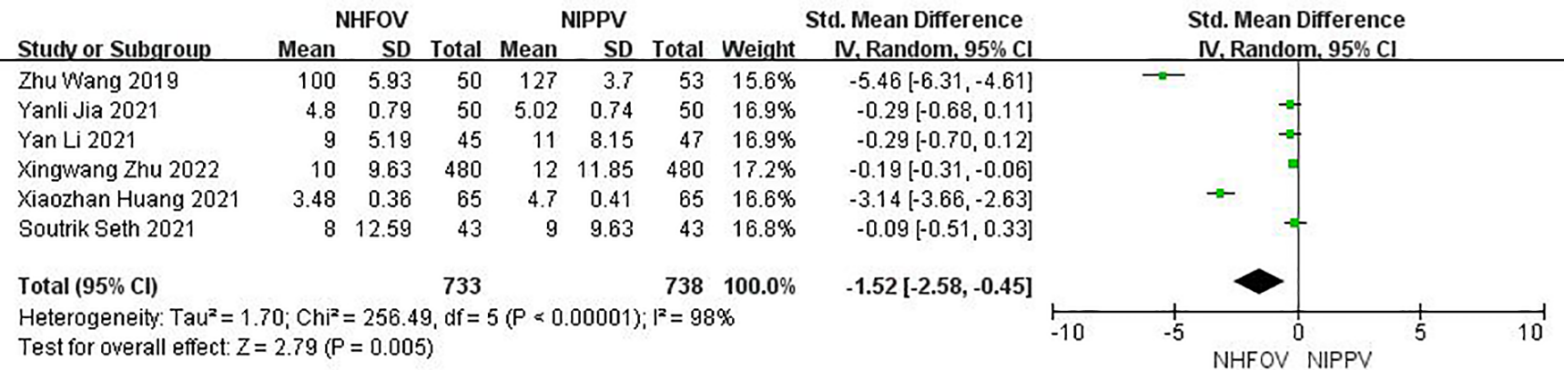
Results of the meta-analysis of duration of non-invasive ventilation.

Six RCTs (12–14, 16–18) that enrolled 1,471 neonates reported the duration of non-invasive ventilation, and found a significant decrease in the duration of non-invasive ventilation using NHFOV (standard mean difference (SMD) = −1.52, 95% CI −2.58, −0.45, *I*^2^ = 98%, *P* = 0.005; very low-quality evidence; Figure 4).

**Figure 5:**
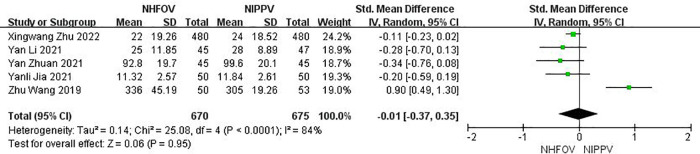
Results of the meta-analysis of total oxygen therapy time.

Five RCTs (12, 13, 15, 17, 18) with 1,345 neonates that reported the total oxygen therapy time found no significant differences between the NHFOV and NIPPV groups (SMD = −0.01, 95% CI −0.37, 0.35, *I*^2^ = 84%, *P* = 0.95; low-quality evidence; Figure 5).

**Figure 6:**
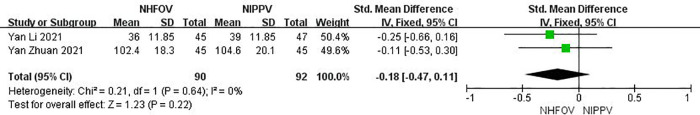
Results of the meta-analysis of length of hospital stay.

Two RCTs (15, 17) with 182 neonates that reported LOS showed no significant difference in the decreased LOS (SMD = −0.18, 95% CI −0.47, 0.11, *I*^2^ = 0%, *P* = 0.22; low-quality evidence; Figure 6).

No significant differences in the likelihood of adverse outcomes were observed, including air leaks (Four RCTs (12, 13, 16, 18), RR = 0.74, 95% CI 0.34, 1.60, *I*^2^ = 36%, *P* = 0.45, low-quality evidence; Supplementary Figure S1), apnea (Two RCTs (13, 18), RR = 0.67, 95% CI 0.33, 1.36, *I*^2 ^= 0%, *P* = 0.26, low-quality evidence; Supplementary Figure S2), abdominal distention (Two RCTs (15, 19), RR = 0.85, 95% CI 0.41, 1.73, *I*^2^ = 0%, *P* = 0.65, low-quality evidence; Supplementary Figure S3), BPD (Six RCTs (12–14, 16–18), RR = 0.88, 95% CI 0.75, 1.02, *I*^2^ = 0%, *P *= 0.09, low-quality evidence; Supplementary Figure S4), NEC (Three RCTs (14, 17, 19), RR = 0.75, 95% CI 0.26, 2.18, *I*^2^ = 0%, *P* = 0.59, low-quality evidence; Supplementary Figure S5), IVH (Four RCTs (14, 16–18), RR = 0.72, 95% CI 0.28, 1,86, *I*^2^ = 0%, *P* = 0.50, low-quality evidence; Supplementary Figure S6), nasal injury (Five RCTs (12, 13, 17–19), RR = 1.01, 95% CI 0.63, 1,63, *I*^2^ = 0%, *P* = 0.96, low-quality evidence; Supplementary Figure S7), ROP (Three RCTs (14, 17, 18), RR = 0.75, 95% CI 0.39, 1,46, *I*^2^ = 0%, *P* = 0.40, low-quality evidence; Supplementary Figure S8), and periventricular leukomalacia (Three RCTs (13, 14, 18), RR = 0.88, 95% CI 0.29, 2.67, *I*^2^ = 0%, *P* = 0.81, low-quality evidence; Supplementary Figure S9).

### Publication bias

We only evaluated publication bias among the reintubation rates, which was the primary outcome parameter. The results suggested that the reintubation rates funnel plots we assessed were symmetrical, and the results of Egger's test were not significant, indicating the absence of publication bias (Supplementary Figure S10).

### Sensitivity analysis

In sensitivity analysis, based on the stepwise omission of one study at a time, we found no significant difference in the duration of non-invasive ventilation in post-extubation respiratory support when the Zhu Wang et. al (18). study was excluded (Supplementary Figure S11). Exclusion of the Zhu Wang et al. (18) study led to reducing the total oxygen therapy time (Supplementary Figure S12). There was no significant change in other outcome parameters.

### The overall quality of the evidence

The evidence was judged to be of low quality of the outcome of total oxygen therapy time, hospitalization time, air leak, abdominal distension, BPD, IVH, ROP, NEC, nasal injury, periventricular leukomalacia, and apnea. Evidence for the outcome of the rate of reintubation and duration of noninvasive ventilation was judged to be very low quality (online Supplementary Table S1).

## Discussion

This meta-analysis of the findings from eight trials, including 1,603 participants compared the efficacy and safety of post-extubation respiratory support for neonates using NHFOV and NIPPV. The results showed that significant difference was found in the reduction of reintubation rates between NHFOV and NIPPV. Compared with NIPPV, NHFOV could decrease the duration of non-invasive ventilation. But there were no significant differences between the two groups for the adverse outcomes. It is worth noting that some of the results were found to have changed in sensitivity analysis, and the overall quality of the evidence of the outcomes was low or very low. Therefore, the results should be interpreted with caution.

Reintubation within 72 h of initial extubation in very low or extremely low birth weight infants was independently associated with increased risk of BPD/death and death in a retrospective cohort study (20). This result is consistent with a multicenter observational study (21) that demonstrated that for preterm infants, reintubation after elective extubation was an independent risk factor for death or moderate to severe BPD, especially reintubation within 48 h of extubation. For a successful transition from invasive to noninvasive ventilation, the mode choice of noninvasive ventilation is critical to avoid reintubation. In our study, the reduction in reintubation favored NHFOV over NIPPV, and the difference between the two groups was statistically significant. The reintubation rates of NHFOV and NIPPV were 11.51% (92/799) and 17.04% (137/804), respectively. However, the analysis should be considered with caution given the heterogeneity of criteria of reintubation and settings of NHFOV among included studies. In the study by Xingwang Zhu (12) and Yan Li et al. (17), the reintubation rates in the NHFOV group were 8.96% and 8.89%, respectively, lower than average, despite their reintubation criteria were broader, including severe respiratory acidosis, hypoxemia, severe apnea, or pulmonary hemorrhage, etc. We believe this discrepancy is caused by the difference of the NHFOV settings. In the study by Xingwang Zhu (12) and Yan Li et al. (17), the amplitude could be adjusted from 25 to 50 cmH_2_O, which is higher than the range of 12 to 16 cmH_2_O and 25 to 35 cmH_2_O specified in other studies. Using lower frequency and higher amplitude in the NHFOV device increases tidal volume and promotes CO_2_ removal (22). In addition, high mean airway pressure (MAP) may also play a role, as low MAP in NHFOV probably failed to recruit the lung effectively. Xingwang Zhu et al. (12) set MAP at 5 to 16 cmH_2_O, and Yan Li et al. (17) set MAP at 6 to 12 cmH_2_O. They were able to adjust MAP to a higher level than other studies that set MAP at 6 to 8 cmH_2_O and 8 to 10 cmH_2_O.

NHFOV holds promise as a non-invasive mode of ventilation that may help reduce the risk of reintubation in selected high-risk patients. Czernik et al. (23) have investigated the feasibility of NHFOV immediately after extubation in preterm infants at high risk of extubation failure. Of the 20 infants,14 remained extubated and were transitioned to another noninvasive ventilation mode, after remaining on NHFOV for a minimum of 32 h. A multiple institution North American Retrospective case series study (24) reported that about 58% of all NHFOV instances (rescue or prophylactic) resulted in a successful transition to another noninvasive ventilation mode, thus preventing intubation in the majority of this high-risk patient group. Wang et al. (25) also suggested that NHFOV can be used as a treatment after failure of other non-invasive assisted ventilation support, or as a preventive treatment for children at high risk of extubation failure, avoiding endotracheal intubation and mechanical ventilation. However, these were relatively small observational studies without a control group, and it is difficult to predict how many infants would have remained extubated even without the use of NHFOV. Due to limitations in the published data, our study was unable to perform subgroup analyses for the preterm infants at high risk, such as less than 28 weeks and less than 1000 g. Future rigorously designed prospective randomized clinical trials are needed to provide definitive answers regarding the role of NHFOV in preterm infants at high risk of extubation.

Besides efficacy, safety was another important focus when NHFOV was used. The European survey (26) described that abdominal distention and upper airway obstruction due to viscous secretions were the most frequently reported side effects of NHFOV. However, these were only surveyed as physicians' opinions. Soutrik Seth et al. (16) did not find such alarming side effect as viscous secretions, which they attributed to good nursing care and maintenance of oral hygiene. And in our study, it was found that there was no significant difference in the risk of abdominal distension between NIPPV and NHFOV.

Ventilator-induced lung injury is a major, potentially modifiable, risk factor implicated in BPD causation. Attenuation of intra-tracheal pressure in NHFOV lowered alveolar pressure, thereby maintaining the end-expiratory volume at a normal level without atelectasis trauma to the lung parenchyma, thus reducing the risk for BPD (27). Our results showed that the reduction of the risk of BPD tended to favor NHFOV over NIPPV, although this result did not reach statistical significance. Fang Zou et al. (28) demonstrated that in the initial respiratory support, compared with NIPPV, NHFOV could reduce the risk of BPD for infants with gestational age less than 31 weeks and birth weight less than 1500 g (23% vs. 41%, *P *<  0.05). Therefore, the benefits of NHFOV in populations at high risk for BPD, especially very preterm and ultra-preterm infants, require further high-quality research.

Undoubtedly, some limitations in our meta-analysis may have affected the interpretation of the findings. Firstly, the trials analyzed the differences in research design and clinical characteristics of the subjects. The heterogeneity in the characteristics of the participants and interventions and the lack of a standardized assessment of reintubation and BPD were additional limitations. Secondly, subgroup analyses based on gestational age or birth weight could not be performed due to the lack of individual patient data. Finally, although the search was not limited by language or publication source, most included studies were from China. We were unable to generalize the current findings to other ethnic groups. The present survey in five European countries showed that neonatologists in 17% of 172 European NICUs used NHFOV for various indications (26), but studies in European-American countries were retrospective case series with relatively small sample sizes.

In conclusion, despite some limitations, this meta-analysis demonstrates that NHFOV could decrease the reintubation rates and shorten the duration of non-invasive ventilation compared with NIPPV. In addition, NHFOV was safe, and no significant difference in the occurrence of adverse outcomes between the two groups. While NHFOV may be of some benefit for post-extubation respiratory support in preterm neonates, definite recommendations cannot be made due to the quality of the published evidence. Further multicenter randomized controlled trials are warranted to recommend using NHFOV as post-extubation respiratory support in the management of preterm neonates.

## Data Availability

The original contributions presented in the study are included in the article/**Supplementary Material**, further inquiries can be directed to the corresponding author/s.
