# Supplementary material for: Use of NHFOV vs. NIPPV for the respiratory support of preterm newborns after extubation: A meta-analysis
Source: Front Pediatr. 2023 Jan 11;10:1063387. doi: 10.3389/fped.2022.1063387 (PMC9874940; doi:10.3389/fped.2022.1063387)
Supplement: Supplementary file 1 [file Table1.docx]

**Supplementary Table 1. Summary of findings for the comparison. GRADE 'Summary of findings' table**

| \| **Patient or population: preterm newborns** \| \| --- \| \| **Intervention: noninvasive high-frequency oscillatory ventilation (NHFOV)** \| \| **Comparison: nasal intermittent positive-pressure ventilation (NIPPV)** \| | | | | | | | | | | | | | |
| --- | --- | --- | --- | --- | --- | --- | --- | --- | --- | --- | --- | --- | --- | --- | --- | --- |
| **Quality assessment** | | | | | | | **No of patients** | | **Effect** | | **Quality** | **Importance** |  |
|  |  |  |  |  |  |  |  |  |  |  |  |  |  |
| **No of studies** | **Design** | **Risk of bias** | **Inconsistency** | **Indirectness** | **Imprecision** | **Other considerations** | **NHFOV** | **NIPPV** | **Relative (95% CI)** | **Absolute** |  |  |  |
| **Rate of reintubation** | | | | | | | | | | | | |  |
| 8 | randomised trials | serious^1^ | serious^3^ | no serious indirectness | serious^2^ | none | 97/799  (11.5%) | 137/804 (17%) | RR 0.64 (0.44 to 0.92) | 61 fewer per 1000 (from 14 fewer to 95 fewer) | ⊕OOO VERY LOW |  |  |
|  |  |  |  |  |  |  |  | 26% |  | 94 fewer per 1000 (from 21 fewer to 146 fewer) |  |  |  |
| **Rate of reintubation - within 72h** | | | | | | | | | | | | |  |
| **5** | randomised trials | serious^1^ | serious^4^ | no serious indirectness | serious^2^ | none | 27/209  (12.9%) | 57/212  (26.9%) | RR 0.51 (0.33 to 0.77) | 132 fewer per 1000 (from 62 fewer to 180 fewer) | ⊕OOO VERY LOW |  |  |
|  |  |  |  |  |  |  |  | 32% |  | 157 fewer per 1000 (from 74 fewer to 214 fewer) |  |  |  |
| **Rate of reintubation - within 7 days** | | | | | | | | | | | | |  |
| 2 | randomised trials | serious^1^ | serious^5^ | no serious indirectness | serious^2^ | none | 22/110  (20%) | 25/112  (22.3%) | RR 0.74 (0.19 to 2.89) | 58 fewer per 1000 (from 181 fewer to 422 more) | ⊕OOO VERY LOW |  |  |
|  |  |  |  |  |  |  |  | 22.8% |  | 59 fewer per 1000 (from 185 fewer to 431 more) |  |  |  |
| **Duration of noninvasive ventilation** | | | | | | | | | | | | |  |
| 6 | randomised trials | serious^1^ | very serious^2^ | no serious indirectness | no serious imprecision | none | 733 | 738 | - | SMD 1.52 lower (2.58 to 0.45 lower) | ⊕OOO VERY LOW |  |  |
| **Total oxygen therapy time** | | | | | | | | | | | | |  |
| 5 | randomised trials | serious^1^ | serious^9^ | no serious indirectness | no serious imprecision | none | 670 | 675 | - | SMD 0.01 lower (0.37 to 0.37 higher) | ⊕⊕OO LOW |  |  |
| **Hospitalization time** | | | | | | | | | | | | |  |
| 2 | randomised trials | serious^1^ | no serious inconsistency | no serious indirectness | serious^8^ | none | 90 | 92 | - | SMD 0.18 lower (0.47 lower to 0.11 higher) | ⊕⊕OO LOW |  |  |
| **Air leak** | | | | | | | | | | | | |  |
| 4 | randomised trials | serious^1^ | no serious inconsistency | no serious indirectness | serious^2^ | none | 11/623  (1.8%) | 15/626  (2.4%) | RR 0.74 (0.34 to 1.6) | 6 fewer per 1000 (from 16 fewer to 14 more) | ⊕⊕OO LOW |  |  |
|  |  |  |  |  |  |  |  | 3.2% |  | 8 fewer per 1000 (from 21 fewer to 19 more) |  |  |  |
| **Abdominal distension** | | | | | | | | | | | | |  |
| 2 | randomised trials | serious^1^ | no serious inconsistency | no serious indirectness | serious^2^ | none | 11/66 (16.7%) | 13/66  (19.7%) | RR 0.85 (0.41 to 1.73) | 30 fewer per 1000 (from 116 fewer to 144 more) | ⊕⊕OO LOW |  |  |
|  |  |  |  |  |  |  |  | 17% |  | 25 fewer per 1000 (from 100 fewer to 124 more) |  |  |  |
| **NEC** | | | | | | | | | | | | |  |
| 3 | randomised trials | serious^1^ | no serious inconsistency | no serious indirectness | serious^2^ | none | 5/131  (3.8%) | 7/133  (5.3%) | RR 0.75 (0.26 to 2.18) | 13 fewer per 1000 (from 39 fewer to 62 more) | ⊕⊕OO LOW |  |  |
|  |  |  |  |  |  |  |  | 4.8% |  | 12 fewer per 1000 (from 36 fewer to 57 more) |  |  |  |
| **IVH** | | | | | | | | | | | | |  |
| 4 | randomised trials | serious^1^ | no serious inconsistency | no serious indirectness | serious^2^ | none | 7/203  (3.4%) | 10/208  (4.8%) | RR 0.72 (0.28 to 1.86) | 13 fewer per 1000 (from 35 fewer to 41 more) | ⊕⊕OO LOW |  |  |
|  |  |  |  |  |  |  |  | 5.2% |  | 15 fewer per 1000 (from 37 fewer to 45 more) |  |  |  |
| **Nasal injury** | | | | | | | | | | | | |  |
| 5 | randomised trials | serious^1^ | no serious inconsistency | no serious indirectness | serious^2^ | none | 32/646  (5%) | 32/651  (4.9%) | RR 1.01 (0.63 to 1.63) | 0 more per 1000 (from 18 fewer to 31 more) | ⊕⊕OO LOW |  |  |
|  |  |  |  |  |  |  |  | 5.7% |  | 1 more per 1000 (from 21 fewer to 36 more) |  |  |  |
| **BPD** | | | | | | | | | | | | |  |
| 6 | randomised trials | serious^1^ | no serious inconsistency | no serious indirectness | serious^2^ | none | 209/733  (28.5%) | 240/738  (32.5%) | RR 0.88 (0.75 to 1.02) | 39 fewer per 1000 (from 81 fewer to 7 more) | ⊕⊕OO LOW |  |  |
|  |  |  |  |  |  |  |  | 28.8% |  | 35 fewer per 1000 (from 72 fewer to 6 more) |  |  |  |
| **Apnea** | | | | | | | | | | | | |  |
| 2 | randomised trials | serious^1^ | no serious inconsistency | no serious indirectness | serious^2^ | none | 11/100  (11%) | 17/103  (16.5%) | RR 0.67 (0.33 to 1.36) | 54 fewer per 1000 (from 111 fewer to 59 more) | ⊕⊕OO LOW |  |  |
|  |  |  |  |  |  |  |  | 16.4% |  | 54 fewer per 1000 (from 110 fewer to 59 more) |  |  |  |
| **ROP** | | | | | | | | | | | | |  |
| 3 | randomised trials | serious^1^ | no serious inconsistency | no serious indirectness | serious^2^ | none | 13/160  (8.1%) | 18/165  (10.9%) | RR 0.75 (0.39 to 1.46) | 27 fewer per 1000 (from 67 fewer to 50 more) | ⊕⊕OO LOW |  |  |
|  |  |  |  |  |  |  |  | 9.4% |  | 23 fewer per 1000 (from 57 fewer to 43 more) |  |  |  |
| **Periventricular leukomalacia** | | | | | | | | | | | | |  |
| 3 | randomised trials | serious^1^ | no serious inconsistency | no serious indirectness | serious^2^ | none | 6/165  (3.6%) | 7/168  (4.2%) | OR 0.88 (0.29 to 2.67) | 5 fewer per 1000 (from 29 fewer to 62 more) | ⊕⊕OO LOW |  |  |
|  |  |  |  |  |  |  |  | 5.7% |  | 6 fewer per 1000 (from 40 fewer to 82 more) |  |  |  |

^1^ Downgraded one level for serious risk of bias: lack of blinding could influence outcomes
^2^ Downgraded one level for serious imprecision: limited number of trials; low event rates

^3^ Downgraded one level for serious Inconsistency: heterogeneity in results I2 = 73%

^4^ Downgraded one level for serious Inconsistency: heterogeneity in results I2 = 79%

^5^ Downgraded one level for serious Inconsistency: heterogeneity in results I2 = 80%

^6^ Downgraded two level for serious Inconsistency: heterogeneity in results I2 = 98%
^7^ Downgraded two level for serious Inconsistency: heterogeneity in results I2 = 97%

^8^ Downgraded one level for serious imprecision: limited number of trials

^9^ Downgraded two level for serious Inconsistency: heterogeneity in results I2 = 88%
